# The Density of Knobs on *Plasmodium falciparum*-Infected Erythrocytes Depends on Developmental Age and Varies among Isolates

**DOI:** 10.1371/journal.pone.0045658

**Published:** 2012-09-20

**Authors:** Katharina A. Quadt, Lea Barfod, Daniel Andersen, Jonas Bruun, Ben Gyan, Tue Hassenkam, Michael F. Ofori, Lars Hviid

**Affiliations:** 1 Centre for Medical Parasitology at i, Faculty of Health and Medical Sciences, University of Copenhagen and at Department of Infectious Diseases, Copenhagen University Hospital (Rigshospitalet), Copenhagen, Denmark; 2 Department of Immunology, Noguchi Memorial Institute for Medical Research, University of Ghana, Legon, Ghana; 3 Nano-Science Centre, Department of Chemistry, Faculty of Science, University of Copenhagen, Copenhagen, Denmark; University of Heidelberg Medical School, Germany

## Abstract

**Background:**

The virulence of *Plasmodium falciparum* malaria is related to the parasite’s ability to evade host immunity through clonal antigenic variation and tissue-specific adhesion of infected erythrocytes (IEs). The *P. falciparum* erythrocyte membrane protein 1 (PfEMP1) family expressed on dome-shaped protrusions called knobs on the IE surface is central to both. Differences in receptor specificity and affinity of expressed PfEMP1 are important for IE adhesiveness, but it is not known whether differences in the number and size of the knobs on which the PfEMP1 proteins are expressed also play a role. Therefore, the aim of this study was to provide detailed information on isolate- and time-dependent differences in knob size and density.

**Methodology/Principal Findings:**

We used atomic force microscopy to characterize knobs on the surface of *P. falciparum*-infected erythrocytes. Fourteen *ex vivo* isolates from Ghanaian children with malaria and 10 *P. falciparum* isolates selected *in vitro* for expression of a particular PfEMP1 protein (VAR2CSA) were examined. Knob density increased from ∼20 h to ∼35 h post-invasion, with significant variation among isolates. The knob density *ex vivo*, which was about five-fold higher than following long-term *in vitro* culture, started to decline within a few months of culture. Although knob diameter and height varied among isolates, we did not observe significant time-dependent variation in these dimensions.

**Conclusions/Significance:**

The density of knobs on the *P. falciparum*-IE surface depends on time since invasion, but is also determined by the infecting isolate in a time-independent manner. This is the first study to quantitatively evaluate knob densities and dimensions on different *P. falciparum* isolates, to examine *ex vivo* isolates from humans, and to compare *ex vivo* and long-term *in vitro*-cultured isolates. Our findings contribute to the understanding of the interaction between *P. falciparum* parasites and the infected host.

## Introduction

*P. falciparum* is the parasite causing the most virulent form of malaria in humans, and the parasite responsible for the large majority of severe cases and malaria-related deaths. In 2010 there were about 216 million clinical cases and about 655,000 malaria deaths [1]. The high virulence of *P. falciparum* is related to the characteristic accumulation of late stage-IEs in various tissues, which interferes with splenic clearance of IEs and can lead to development of high parasitemias and life-threatening inflammation and circulatory disturbances [2], [3]. This ability to sequester is related to the expression of characteristic parasite-encoded dome-shaped knob structures on the IE surface [4], [5]. Although similar excrescences have been reported on the surface of erythrocytes infected by many species of malaria parasites and other apicomplexan parasites [6]–[9], only the knobs of *P. falciparum* are known to display specific adhesins. These adhesins are members of the protein family *P. falciparum* erythrocyte membrane protein 1, PfEMP1 [10]) that are capable of mediating high-affinity interaction with a range of host vascular receptors. The PfEMP1 proteins are therefore central to the pathogenesis of *P. falciparum* malaria, important targets of naturally acquired immunity, and considered by many to be promising candidates for vaccination against the disease [11], [12]. As a consequence, there is a large body of literature on most aspects of the PfEMP1 and the *var* genes that encode them. In contrast, surprisingly little is known about the dynamics of the IE surface knobs on which these antigens are displayed. The limited data available indicate that the density of these knobs increases as the intra-erythrocytic parasites mature [13], [14], but it is not known if it varies among isolates or are influenced by the particular PfEMP1 protein displayed, and no information regarding *ex vivo* parasites from malaria patients exists. In this study, we used atomic force microscopy (AFM) [15] as a convenient nano-scale resolution technique to address these issues.

## Results

### The Density of Knobs on the Surface of P. falciparum-infected Erythrocytes Increases during the First Part of the Asexual Replication Cycle

**Figure 1:**
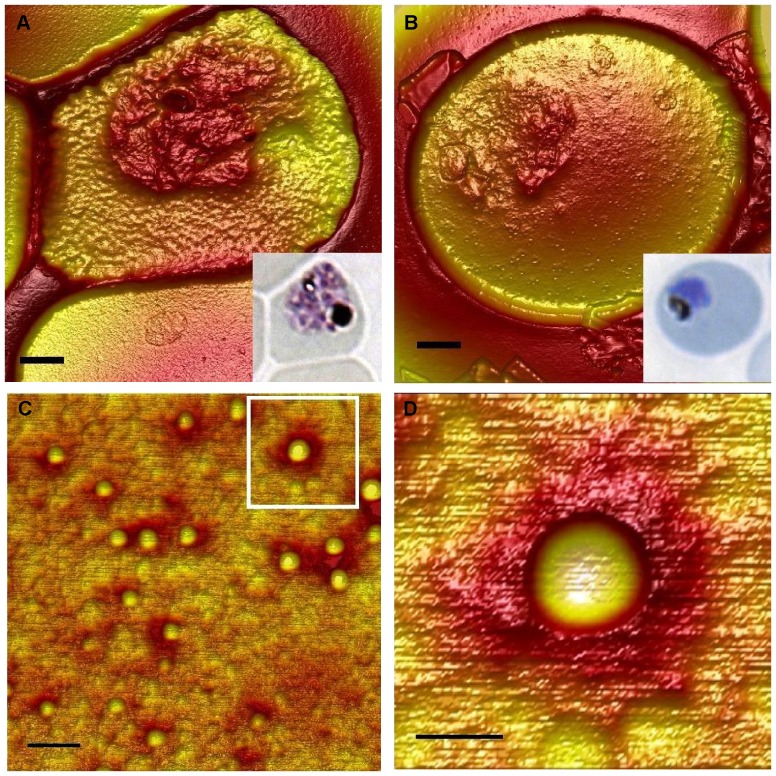
Parasite developmental age and infected erythrocyte knob density. Example atomic force micrographs of erythrocytes infected by a early schizont parasite *ex vivo* (A) or by a younger VAR2CSA-expressing trophozoite (B). Black scale bars: 1 µm. The inserts show light micrographs of the same infected erythrocytes stained by Giemsa. Close-up micrographs of knobs (C) and of a single knob (D). Black scale bars: 200 nm (C), 75 nm (D).

**Figure 2:**
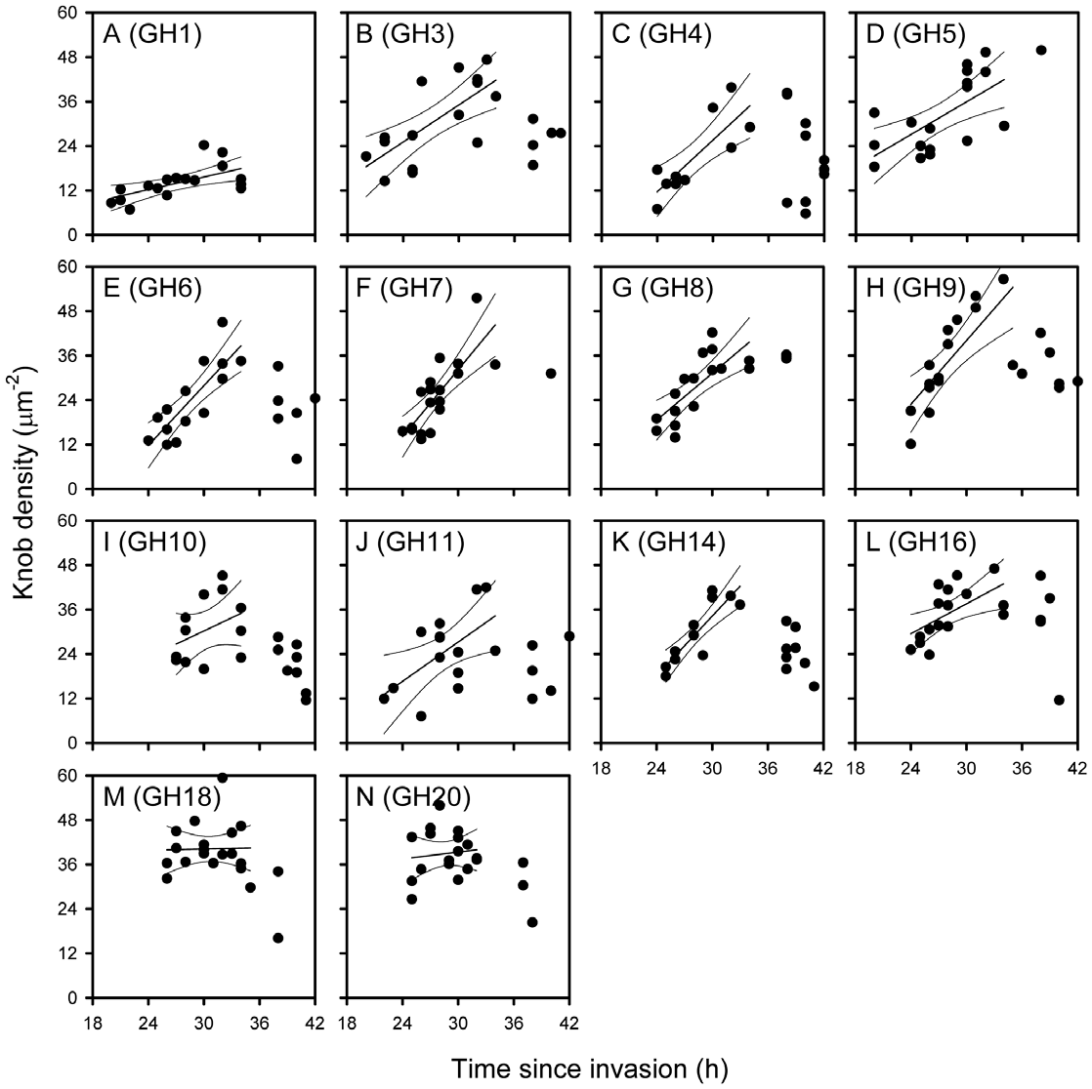
The density of knobs on the surface of erythrocytes infected by ex vivo isolates of *P. falciparum* obtained from Ghanaian acute malaria patients. The relationship between time since invasion (h) and IE surface knob density (µm^–2^) on erythrocytes infected by 14 genotypically distinct isolates of *P. falciparum* (isolate name in brackets), cultured *in vitro* for less than 30 h (A-N). Individual data points, as well as the linear regression line (with 95% confidence limits) for data points <36 h are shown for each isolate.

We first studied 14 isolates obtained from Ghanaian pediatric *P. falciparum* malaria patients. As the young IEs in the circulation of malaria patients are knob-negative, we cultured the IEs briefly (about 20 h) *in vitro* to obtain knob-expressing IEs (Fig. 1) with a development age of approximately 20–40 h. The time/density scatter plots generally showed increasing knob densities with time until approximately 36 h after invasion, followed by decreasing densities for the remainder of the cycle (Fig. 2). This non-linear relationship was supported by statistical analysis, which showed significant linear regression slopes in only three isolates (GH5, GH7, and GH8), with highly significant departures from linearity in seven, including GH8 (Table S1). Thus, a significant linear knob density/time relation was seen in just two isolates (GH5 and GH7), and it is noteworthy that only a single data-point >35 h was available for either of those. However, when we re-analyzed the data after excluding all time points later than 36 h, we found significant linear knob density/time since invasion relationships for all the isolates except three (GH10, GH18, and GH20), and no significant departures from linearity for any of the isolates (Fig. 2 and Table S2). The lack of significant knob density/time relationship for GH10, GH18, and GH20 could well be due to an insufficient number of early time points for those three isolates (Fig. 2I, M, N). We conclude that the knob density on the surface of IEs increases linearly until about 36 h after invasion, followed by a decline in knob density towards IE rupture.

### The Infected Erythrocyte Knob Density Varies Among Isolates Independent of Time Since Invasion

**Figure 3:**
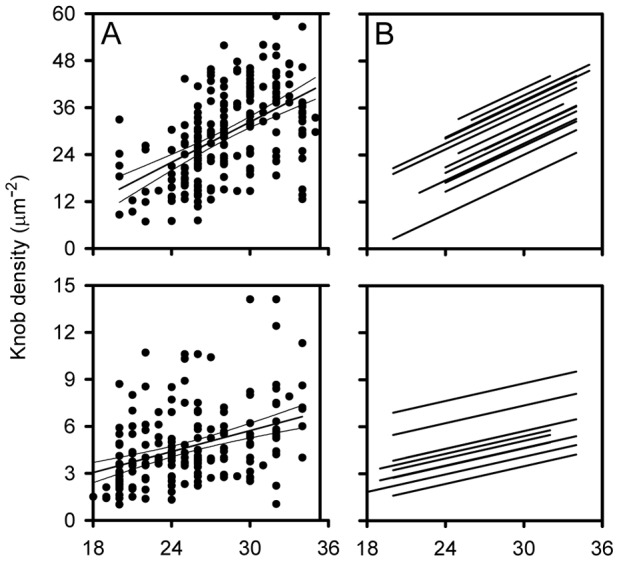
Covariance analysis of the relationship between knob density and time since invasion. All time/density data points <36 h as well as the common regression line (with 95% confidence interval) for the Ghanaian *ex vivo* isolates (A) and the laboratory isolates selected for VAR2CSA expression *in vitro* (C). Adjusted regression lines (assuming parallelism) of individual Ghanaian (B) and VAR2CSA-expressing isolates (D).

In addition to the time-dependent differences described above, the estimated individual regression lines for IEs <36 h indicated that the densities of knobs on the IE surface also varied among the isolates independent of time (Fig. 2). To examine this hypothesis, we first tested whether the differences among the estimated regression line slopes were statistically significant (i.e., whether the lines should be considered non-parallel). On the one hand, the slope of the common regression line for time-points <36 h was highly significant (P(F)<0.005) (Fig. 3A), supporting the above conclusion of an age-dependent increase in knob density. On the other hand, the variance ratio for the individual regression line slopes was not significant (P(F)≥0.05), and the individual regression lines could therefore reasonably be considered parallel (Fig. 3B). In other words, the rate of change in knob density among these isolates appeared to depend only on time since reinvasion. On this basis we evaluated the differences in the relative position of the regression lines, to test the hypothesis that knob density also varied among the isolates independently of age. Covariance analysis confirmed this hypothesis (P(F)<0.005). We conclude that the density of knobs on the surface of *P. falciparum*-infected erythrocytes vary significantly among isolates independent of time.

### The Density of Knobs on the Surface of P. falciparum-infected Erythrocytes Varies Among Isolates Expressing the Same PfEMP1 Protein

**Figure 4:**
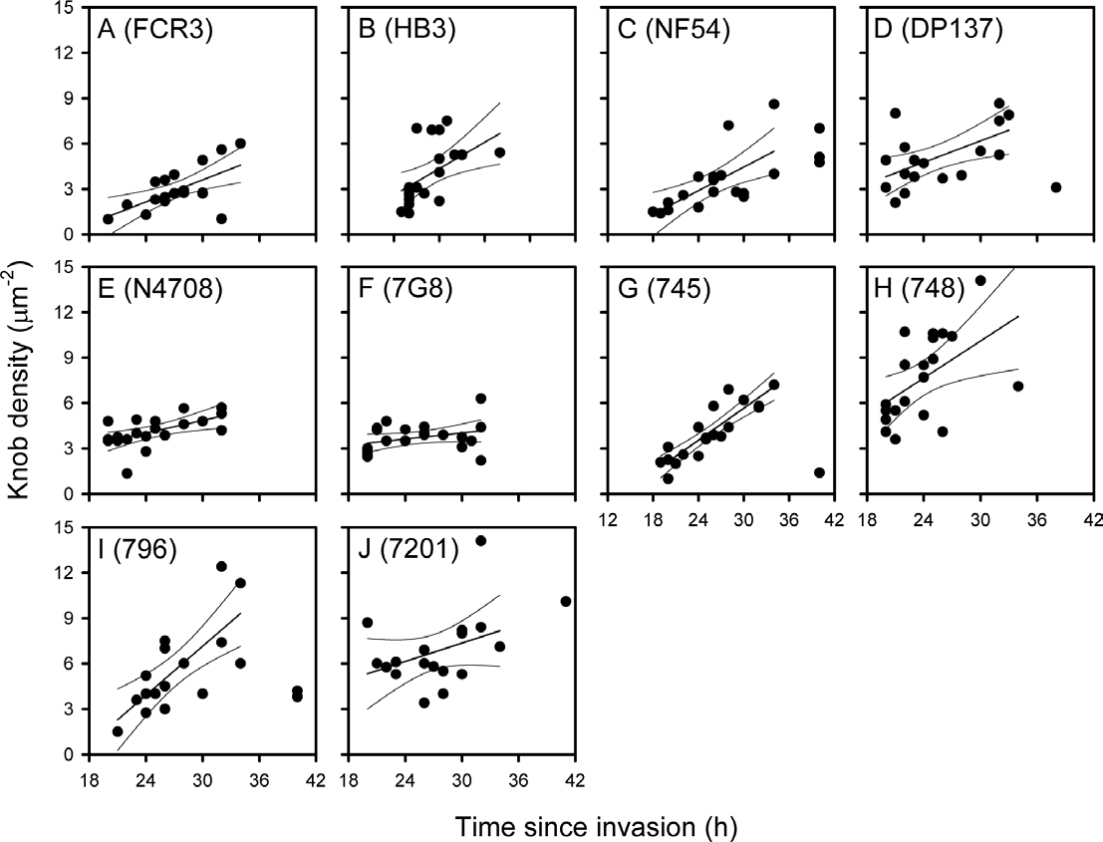
The density of knobs on the surface of erythrocytes infected by long-term in vitro isolates of *P. falciparum* expressing the PfEMP1 protein VAR2CSA. The relationship between time since invasion (h) and IE surface knob density (µm^–2^) on erythrocytes infected by 10 genotypically distinct isolates of *P. falciparum* (isolate name in brackets), maintained in long-time *in vitro* culture and selected for expression of the PfEMP1 protein VAR2CSA by regular panning for IE adhesion to CSA (A-J). Individual data points, as well as the linear regression line (with 95% confidence limits) for data points <36 h are shown for each isolate.

It is highly unlikely that all the Ghanaian *P. falciparum* isolates described above expressed the same or even similar PfEMP1 proteins. The above results therefore raised the possibility that the observed variation in knob density was influenced by the PfEMP1 proteins expressed on the IE surface knobs of the different isolates. To address this possibility, we next studied 10 different long-term *in vitro*-cultured isolates that had all been selected to express the same PfEMP1 protein (VAR2CSA) by repeated panning on the VAR2CSA adhesion receptor chondroitin sulfate A (CSA). As for the Ghanaian isolates, the density of knobs increased in a linear fashion for the first 36 h of the intraerythrocytic cycle (Fig. 4). Increases were statistically significant for 9 of the isolates, and no significant departures from linearity were observed (Table S3). Furthermore, the slope of the common regression line was again highly significant (P(F)<0.005) (Fig. 3C), and the individual regression lines could be considered parallel (Fig. 3D). There was significant variation among the position of the regression lines (P(F)<0.005), and we therefore conclude that the variation in the density of IE surface knobs among isolates does not depend only on the PfEMP1 protein expressed.

### Knob Densities Decrease after Long-term in vitro Cultures

**Figure 5:**
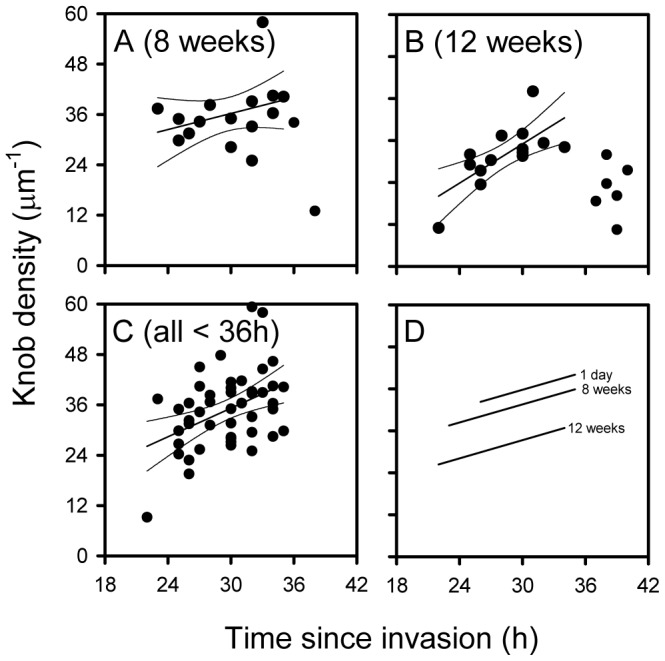
The density of knobs on the surface of erythrocytes infected by isolate GH18 after prolonged culture *in vitro*. The relationship between time since invasion and IE surface knob density after *in vitro* culture of isolate GH18 for 8 weeks (A) or 12 weeks (B). All time/density data points <36 h as well as the common regression line (with 95% confidence interval) for the GH18 (note that the *ex vivo* data points shown in Fig. 2M are included) (C). Individual data points, as well as the linear regression line (with 95% confidence limits) for data points <36 h are shown. Adjusted regression lines (assuming parallelism) after different times of *in vitro* culture of isolate GH18 (D).

The knob densities among *ex vivo* isolates (range 9 /µm^2^ to 32 /µm^2^ at 24 h) were much higher than among the *in vitro*-maintained, VAR2CSA-positive isolates (range 2 /µm^2^ to 8 /µm^2^ at 24 h), as was the rate of increase in knob density with time (common slopes: 1.6 /µm^2^/h vs. 0.2 /µm^2^/h). This is in broad agreement with earlier studies showing reduced knob expression following long-term culture *in vitro* [16]. We therefore kept one of the Ghanaian isolates (GH18) in *in vitro* culture for 12 weeks to see whether the knob density would decline appreciably during the period. Using the same approach as above, we could show that knob densities after 2 days, 8 weeks, and 12 weeks were significantly different from each other, despite regular selection for maintenance of knobs (Fig. 2M, Fig. 5 and Table S4). We conclude that the low knob densities among the VAR2CSA-expressing parasites are related to their long time in culture *in vitro* rather than to their expression of a particular PfEMP1 protein.

### Knob Dimensions do not Vary Significantly during the Intra-erythrocytic Multiplication Cycle

As mentioned above, we found that the knob densities declined during the final twelve hours of the intra-erythrocytic multiplication cycle (Fig. 2). The authors of similar earlier data [17] proposed that this may be due either to sloughing or to coalescing of knob material, and noted that knobs were often larger on older than on younger IEs. We therefore measured the height and diameter of the dome-shaped knobs (Fig. 1C–D) on the Ghanaian as well as on the VAR2CSA-positive isolates. Among the Ghanaian *ex vivo* isolates, only two showed a statistically significant relationship between knob diameter and time since invasion (downwards for GH6 and upwards for GH20) (Fig. S1 and Table S5). The overall mean diameter was 64 nm (±12 nm; S.D.), although the diameters of individual isolates varied significantly (P(F)<0.001), ranging from 50±7 nm (GH18) to 79±12 nm (GH11). Two isolates showed significant relationships between knob height and time since invasion (downward for GH6 and upward for GH8) (Fig. S2 and Table S6). The overall average knob height was 2.9±1.7 nm, but varied significantly among the isolates (P(F)<0.001) from 1.9±1.1 nm (GH9) to 5.1±2.3 nm (GH11).

Among the isolates selected for VAR2CSA expression, only N4708 showed a significant diameter/time relationship (Fig. S3 and Table S7). At 80 nm (±25 nm; S.D.), the overall mean diameter was higher than that of the Ghanaian isolates, with significant variation (P(F)<0.001) among isolates (range from 71±16 nm (HB3) to 101±38 nm (N4708). Three isolates showed significant positive relationships between knob height and time since invasion; two upwards (FCR3 and N4708), one downward (748) (Fig. S4 and Table S8). The overall average knob height was 4.4±2.5 nm, but varied significantly among the isolates (P(F)<0.001) from 3.1±1.5 nm (NF54) to 7.2±3.0 nm (748).

Overall, we did not detect systematic variation in knob dimensions during the intra-erythrocytic development cycle of any given isolate, although there is significant variation among isolates.

## Discussion

In this study, we used AFM to measure at different time points the knob density on the surface of erythrocytes infected by *ex vivo* parasites from patients, and by parasites maintained and selected *in vitro* to express the same PfEMP1 protein. Our objective was to investigate whether knob densities vary among isolates or are influenced by the particular PfEMP1 protein displayed on them, as well as to provide information regarding knob expression on IEs *ex vivo* from malaria patients.

We observed a steadily increasing IE surface knob density 20–35 h post-invasion, followed by a decline towards the end of the intra-erythrocytic cycle. This fits some earlier microscopy data [13], [18], although others have reported steadily increasing densities throughout the cycle [14]. Our data are furthermore supported by molecular evidence. Thus, transcription of parasite genes encoding factors involved in parasite-host interactions such as the *var* genes peaks about 12 h after invasion and is rapidly down-regulated afterwards [19]. However, knobs and the PfEMP1 proteins encoded by the *var* genes do not appear on the IE surface until several hours later [5], [20], [21], with levels reaching a plateau about 10 h after synthesis, i.e., about 24 h after invasion [22]. Export of PfEMP1 to the IE surface appears to have stopped by 30–36 h post-invasion, and knob material such as PfEMP1 is not recycled by the intracellular parasite [22]. Our observation of a decline in knob density about that time therefore indicates that knob density is a function of the parasite export of knobs to the IE surface minus a continuous and steady loss of knobs from the IE surface. Although the IE surface membrane becomes increasingly distorted as the parasite matures inside, which makes knob analysis by AFM more difficult, we find this fact an unlikely explanation for our finding of declining knob densities toward the end of the intra-erythrocytic cycle.

Previously, *P. falciparum*-IE surface knob dimensions have been variously reported to decrease [13] or increase [17] towards the end of the intraerythrocytic cycle. The latter observation was interpreted as evidence that knobs coalescing may account for the late decline in knob density, but that hypothesis is not supported by our data or those of others [14] that knob diameters are relatively stable with time.

The presence of IE surface knobs appears to be critically important for parasite survival *in vivo*, probably facilitating IE adhesion to avoid splenic destruction [23]. We found that IEs *ex vivo* had substantially higher knob densities than erythrocytes infected by *in vitro*-cultured parasites. Furthermore, quite low knob densities on the surface of *in vitro*-propagated parasites have previously been reported [18]. Both sets of observations support the paradigm that knob expression is driven by an *in vivo* selection pressure that is generally absent *in vitro*, although *in vitro* culture conditions might also affect knob expression directly. Complete loss of IE surface knobs after prolonged *in vitro* culture has been observed previously [16], where large proportions of IEs were devoid of knobs after 18–33 months in culture. Such ablation is probably often the result of deletion of genes encoding key structural components of knobs, such as KAHRP at the subtelomeric end of chromosome 2 [24]. Obviously, this may take a long time to occur spontaneously. However, our data indicate that a reduction in knob density can be evident after just a few weeks in culture.

Even *ex vivo* there was substantial variation in knob density among different isolates, but the functional significance of this variation remains unclear. It is tempting to speculate that knob density might correlate with disease severity, and such a correlation has been reported previously for the related parasite *Babesia bovis* (a bovine hemo-parasite also displaying IE sequestration) [9]. Whether it applies to *P. falciparum* must await studies of *ex vivo* parasites from patients with uncomplicated versus severe malaria. In any case, the relation between knob density and *P. falciparum*-IE adhesiveness is not straightforward, as both non-adherent but knobby IEs [25], [26] as well as the opposite [27] have been reported. It thus appears that knobs can be devoid of PfEMP1, while PfEMP1 expression can also persist in the absence of knobs [28]. IEs lose their adhesiveness following splenectomy, and erythrocytes infected by mature parasites can be seen in the circulation although they retain their knobs [25], [26]. This points to a rapid shut-down of *var* transcription and PfEMP1 expression in response to splenectomy, supported by a recent study [29], while reduction and eventual loss of knob expression may follow only later [30].

Our data that genotypically distinct parasites that all expressed the same PfEMP1 protein (VAR2CSA) varied considerably in the density of knobs at the IE surface could be a sign of an intrinsic (PfEMP1- and culture condition-independent) component to the observed diversity. However, it is currently unknown whether the IE knob density of a given parasite clone is affected by selection for surface expression of specific PfEMP1 proteins. Similarly, the significance (if any) of the somewhat larger knobs of the *in vitro*-cultured compared to *ex vivo* isolates remains entirely unclear. It has previously been shown that different hemoglobinopathies affect knob expression and morphology, with grossly abnormal knobs seen on infected α-thalassemic erythrocytes and on infected erythrocytes containing hemoglobin AC, AS, CC, or SS [31]–[33]. It is not known whether knob density and morphology is similarly affected by other characteristics that have also been linked to protection from *P. falciparum* malaria (e.g., blood group antigens).

In conclusion, we have provided the first comprehensive quantitative analysis of data regarding *P. falciparum*-encoded knobs on the IE surface, including the first data on parasite isolates *ex vivo* and isolates selected *in vitro* for expression of the same PfEMP1 protein. We show an initial increase in knob density with time since invasion followed by a decrease coinciding with the cessation of parasite export of knob-associated proteins. Furthermore, we show that there is significant time- and PfEMP1-independent variation in IE surface knob densities among isolates. These data underpin the importance of knob-associated antigens in *P. falciparum* malaria pathogenesis and immunity, although many questions continue to be unanswered.

## Materials and Methods

### Plasmodium Falciparum-infected Erythrocytes

Fourteen different *P. falciparum* isolates obtained from Ghanaian children with acute malaria were studied *ex vivo* (less than 30 h culture *in vitro* after collection by venipuncture to allow study of IEs 16–46 h post invasion). We furthermore examined ten genotypically different *P. falciparum* isolates maintained in long-term *in vitro* culture and selected for adhesion to chondroitin sulfate A (CSA) as described in detail elsewhere [34], [35]. Briefly, these latter parasites were grown in O Rh^+^ erythrocytes in AlbuMax-complemented RPMI 1640 medium, and selected for CSA adhesion and VAR2CSA expression by repeated panning on the placental choriocarcinoma cell line BeWo [36]. At the time of analysis, all these isolates uniformly expressed VAR2CSA [37], [38] as verified by flow cytometry and VAR2CSA-specific monoclonal antibodies [39]–[41]. One of the Ghanaian isolates (GH18) was also maintained *in vitro* for several months, using the same method as above, but without selection for CSA adhesion. Instead, this isolate was subjected to regular selection for knob expression as described elsewhere [42]. The Scientific and Technical Committee and the Institutional Review Board of the Noguchi Memorial Institute for Medical Research approved this study. Parasites were collected from patients after parents consented by signing or thumb printing of the approved informed consent forms. Parasites were collected from only those who agreed to allow the use of parasites isolated from their children.

### Microscopy and Data Acquisition

Light microscopy was used to assess the age (time since invasion) of Giemsa-stained IEs on thin smears, using a Olympus CX41 microscope at 1,000× magnification [43], [44]. The same erythrocytes were subsequently imaged (512×512 pixels) with a Dimension 3100 Scanning Probe microscope (Veeco, Santa Barbara, CA) using an Olympus silicon cantilever (42 N/m, 300 kHz), tapping mode, and scan speeds 0.1–1.0 Hz depending on scan size (0.25–15 µm). All the AFM scanning images were recorded, processed, and analyzed using Nanoscope ver. 6 and ver. 10 software (Veeco). For each isolate, images of about 20 single parasite-IEs at different developmental ages were captured and the knob densities and dimensions determined manually.

### Data Analysis

We used linear regression to test the dependency of knob density on time since invasion in individual isolates. The basic assumption of linearity was tested by analysis of variance, using the method described by Armitage and Berry [45], i.e., by dividing the data into three equally sized groups according to the time points (early, mid, and late), and analyzing the knob density data within each group as replicate measurements at a time point corresponding to the mid-point of the time interval. The regression lines for the different isolates were compared by covariance analysis, after first confirming that the assumption of parallel slopes of the regression lines was reasonable [45].

## Supporting Information

Figure S1 **The diameters of knobs on the surface of erythrocytes infected by** ***ex vivo*** **isolates of** ***P. falciparum*** **obtained from Ghanaian acute malaria patients.** The relationship between time since invasion (h) and IE surface knob diameter (nm) on erythrocytes infected by 14 genotypically distinct isolates of *P. falciparum* (isolate name in brackets), cultured *in vitro* for less than 30 h (A–N). Individual data points, as well as the linear regression line (with 95% confidence limits) for data points <36 h are shown for each isolate.
(TIF)

Figure S2 **The heights of knobs on the surface of erythrocytes infected by** ***ex vivo*** **isolates of** ***P. falciparum*** **obtained from Ghanaian acute malaria patients.** The relationship between time since invasion (h) and IE surface knob height (nm) on erythrocytes infected by 14 genotypically distinct isolates of *P. falciparum* (isolate name in brackets), cultured *in vitro* for less than 30 h (A–N). Individual data points, as well as the linear regression line (with 95% confidence limits) for data points <36 h are shown for each isolate.
(TIF)

Figure S3 **The diameters of knobs on the surface of erythrocytes infected by long-term** ***in vitro*** **isolates of** ***P. falciparum*** **expressing the PfEMP1 protein VAR2CSA.** The relationship between time since invasion (h) and IE surface knob diameter (nm) on erythrocytes infected by 10 genotypically distinct isolates of *P. falciparum* (isolate name in brackets), maintained in long-time *in vitro* culture and selected for expression of the PfEMP1 protein VAR2CSA by regular panning for IE adhesion to CSA (A-J). Individual data points, as well as the linear regression line (with 95% confidence limits) for data points <36 h are shown for each isolate.
(TIF)

Figure S4 **The heights of knobs on the surface of erythrocytes infected by long-term** ***in vitro*** **isolates of** ***P. falciparum*** **expressing the PfEMP1 protein VAR2CSA.** The relationship between time since invasion (h) and IE surface knob height (nm) on erythrocytes infected by 10 genotypically distinct isolates of *P. falciparum* (isolate name in brackets), maintained in long-time *in vitro* culture and selected for expression of the PfEMP1 protein VAR2CSA by regular panning for IE adhesion to CSA (A–J). Individual data points, as well as the linear regression line (with 95% confidence limits) for data points <36 h are shown for each isolate.
(TIF)

Table S1 Analysis of variance with test of linearity – Knob density and time since invasion among Ghanaian ex vivo parasite isolates (all time points).
(DOCX)

Table S2 Analysis of variance with test of linearity – Knob density and time since invasion among Ghanaian ex vivo isolates (only time points <36 h).
(DOCX)

Table S3 Analysis of variance with test of linearity – Knob density and time since invasion among VAR2CSA-expressing long-term parasite isolates.
(DOCX)

Table S4 Analysis of variance with test of linearity – Knob density and time since invasion in isolate GH18 cultured in vitro for various length of time (only time points <36 h).
(DOCX)

Table S5 Analysis of variance with test of linearity – Knob diameter and time since invasion among Ghanaian ex vivo parasite isolates (all time points).
(DOCX)

Table S6 Analysis of variance with test of linearity – Knob height and time since invasion among Ghanaian ex vivo parasite isolates (all time points).
(DOCX)

Table S7 Analysis of variance with test of linearity - Knob diameter and time since invasion among VAR2CSA-expressing long-term parasite isolates.
(DOCX)

Table S8 Analysis of variance with test of linearity - Knob height and time since invasion among VAR2CSA-expressing long-term parasite isolates.
(DOCX)
