# Supplementary figures and images for: The Density of Knobs on Plasmodium falciparum-Infected Erythrocytes Depends on Developmental Age and Varies among Isolates
Source: PLoS One. 2012 Sep 20;7(9):e45658. doi: 10.1371/journal.pone.0045658 (PMC3447797; doi:10.1371/journal.pone.0045658)

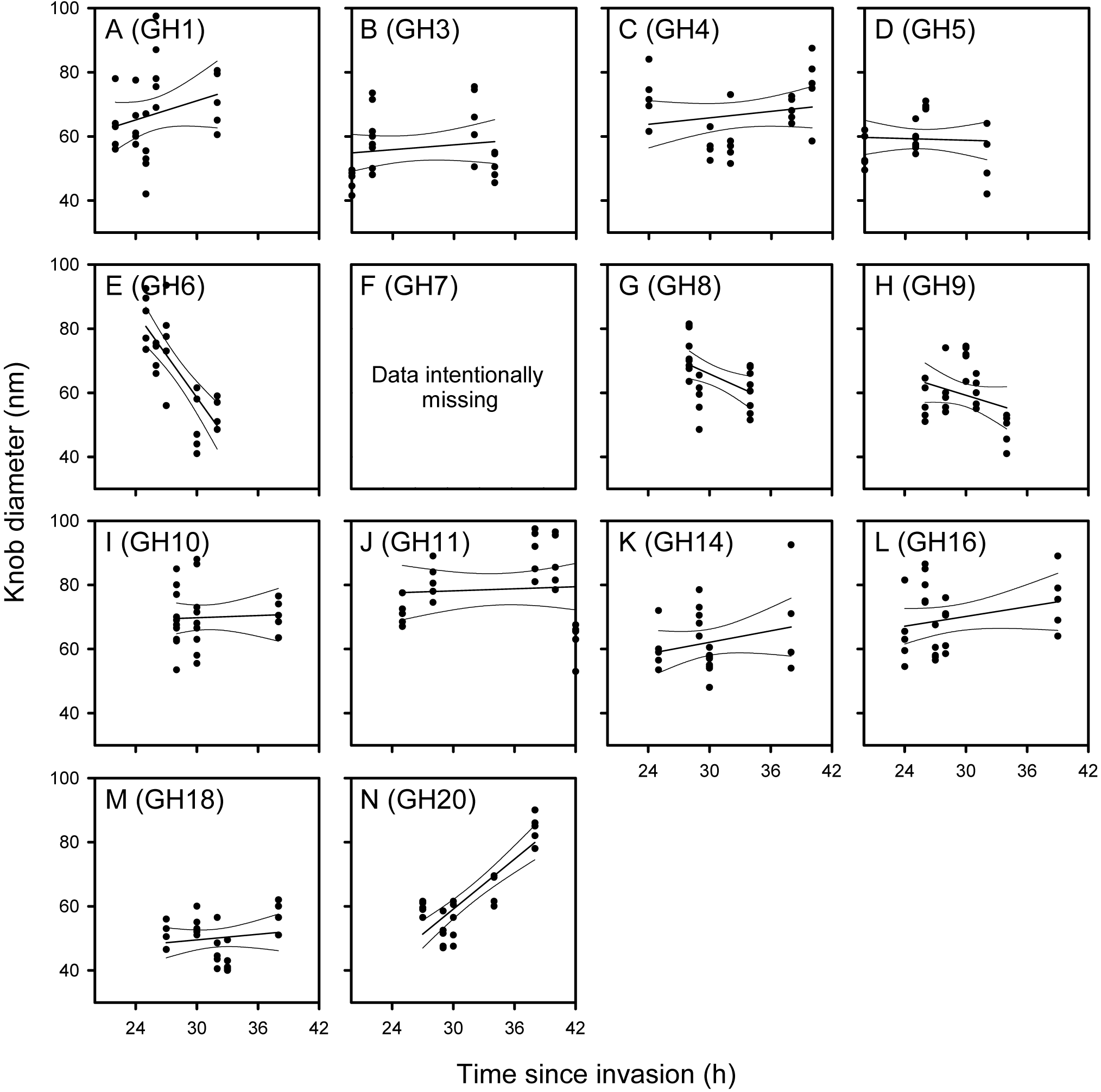

Supplement: Figure S1 — The diameters of knobs on the surface of erythrocytes infected by ex vivo isolates of P. falciparum obtained from Ghanaian acute malaria patients. The relationship between time since invasion (h) and IE surface knob diameter (nm) on erythrocytes infected by 14 genotypically distinct isolates of P. falciparum (isolate name in brackets), cultured in vitro for less than 30 h (A–N). Individual data points, as well as the linear regression line (with 95% confidence limits) for data points <36 h are shown for each isolate. (TIF) [file pone.0045658.s001.tif]

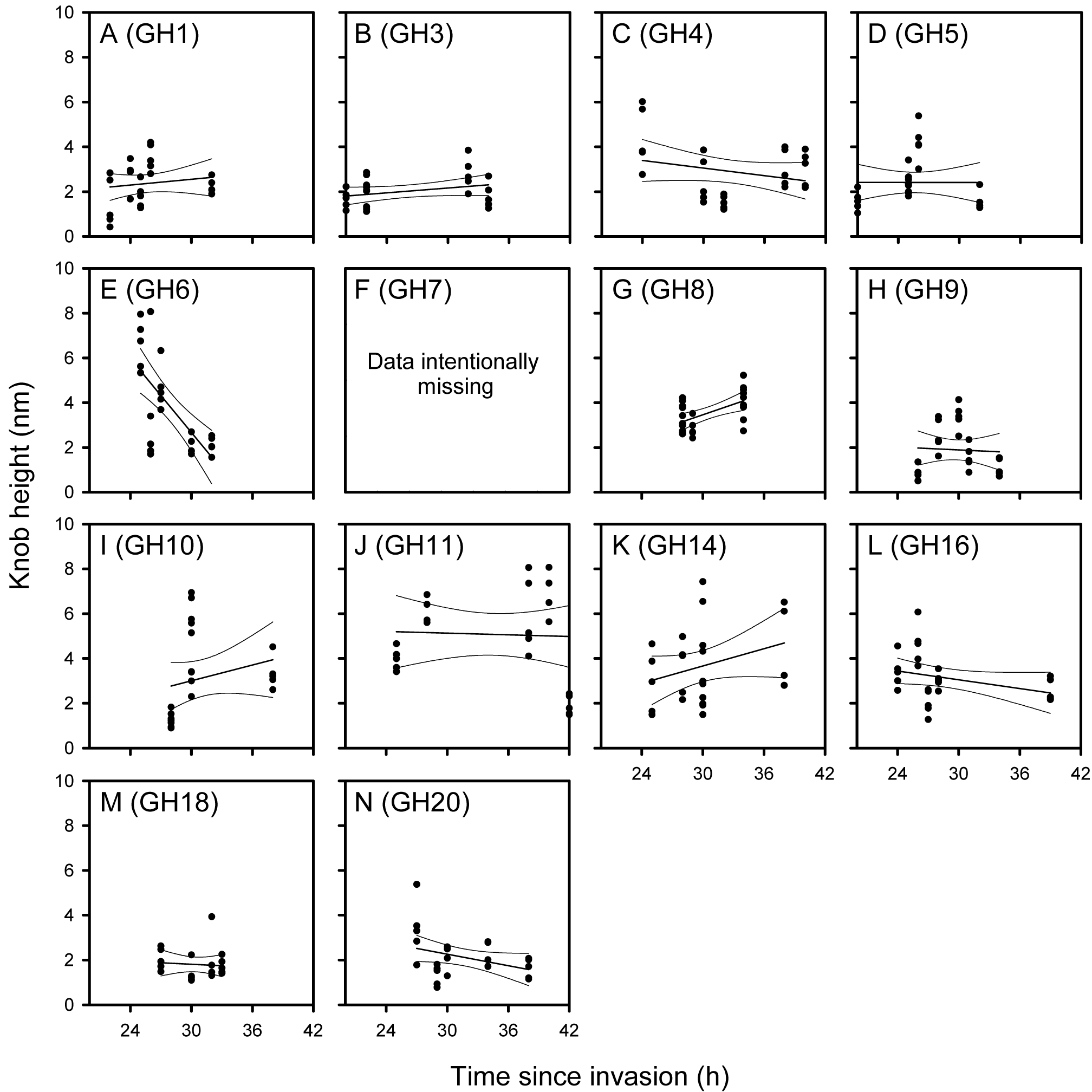

Supplement: Figure S2 — The heights of knobs on the surface of erythrocytes infected by ex vivo isolates of P. falciparum obtained from Ghanaian acute malaria patients. The relationship between time since invasion (h) and IE surface knob height (nm) on erythrocytes infected by 14 genotypically distinct isolates of P. falciparum (isolate name in brackets), cultured in vitro for less than 30 h (A–N). Individual data points, as well as the linear regression line (with 95% confidence limits) for data points <36 h are shown for each isolate. (TIF) [file pone.0045658.s002.tif]

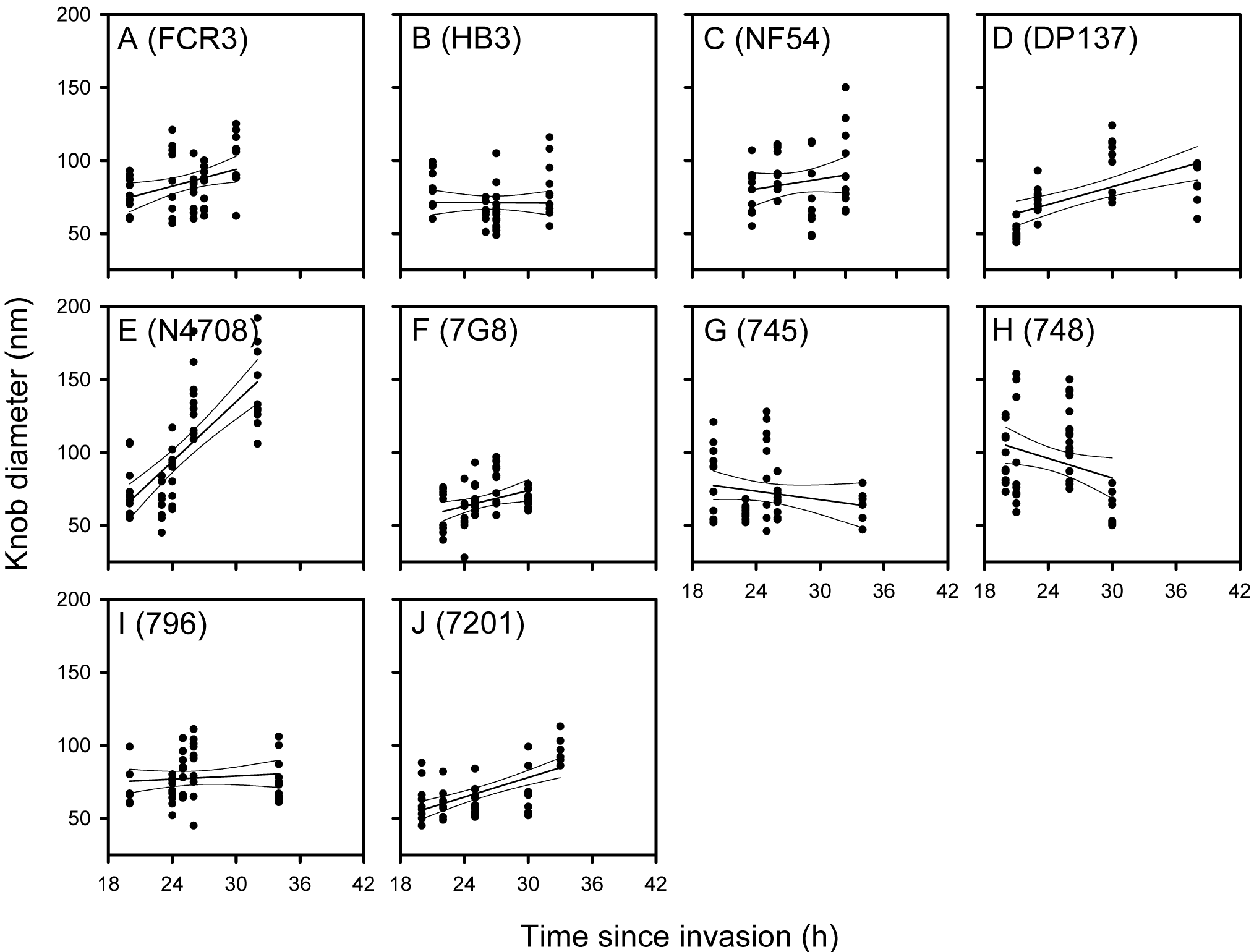

Supplement: Figure S3 — The diameters of knobs on the surface of erythrocytes infected by long-term in vitro isolates of P. falciparum expressing the PfEMP1 protein VAR2CSA. The relationship between time since invasion (h) and IE surface knob diameter (nm) on erythrocytes infected by 10 genotypically distinct isolates of P. falciparum (isolate name in brackets), maintained in long-time in vitro culture and selected for expression of the PfEMP1 protein VAR2CSA by regular panning for IE adhesion to CSA (A-J). Individual data points, as well as the linear regression line (with 95% confidence limits) for data points <36 h are shown for each isolate. (TIF) [file pone.0045658.s003.tif]

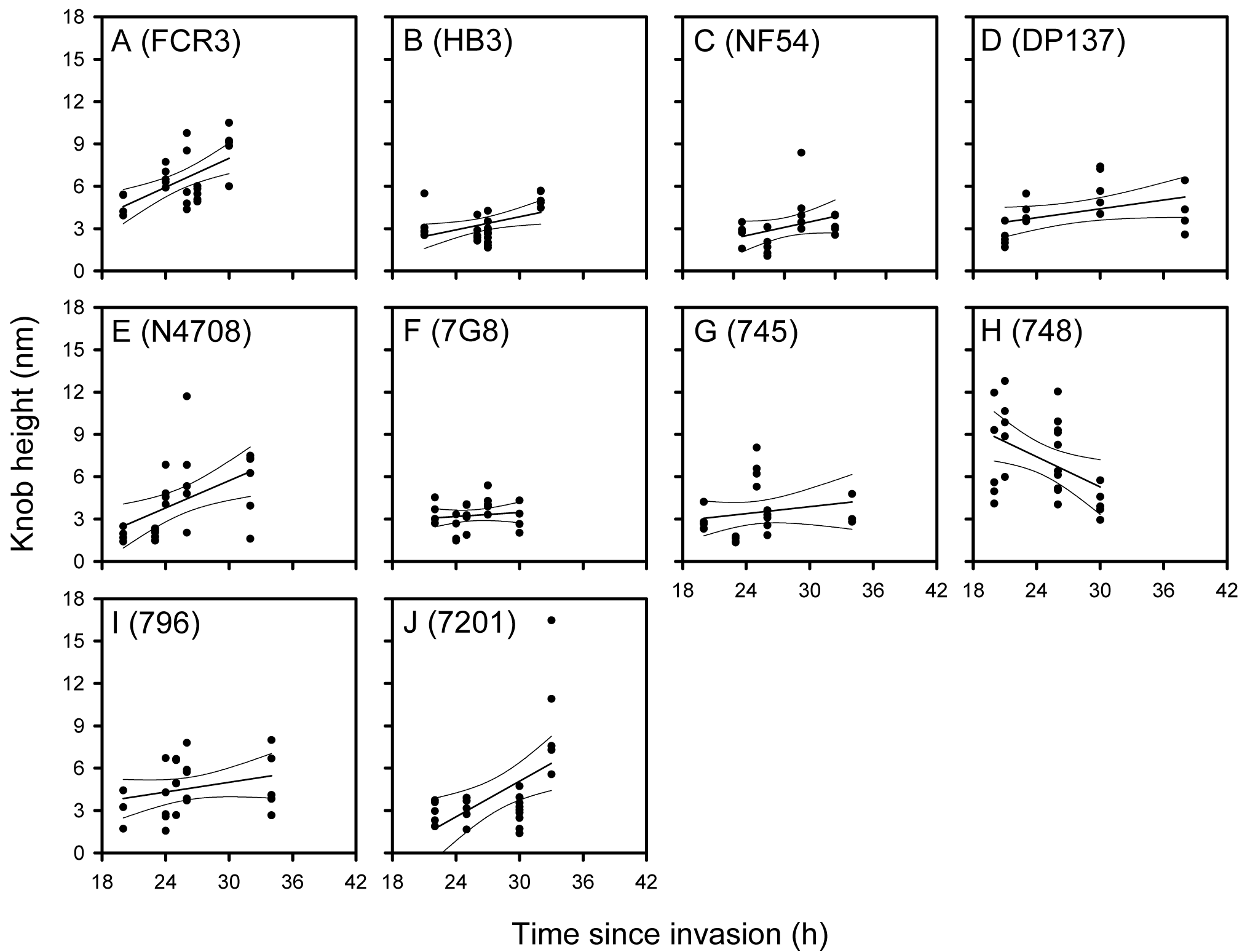

Supplement: Figure S4 — The heights of knobs on the surface of erythrocytes infected by long-term in vitro isolates of P. falciparum expressing the PfEMP1 protein VAR2CSA. The relationship between time since invasion (h) and IE surface knob height (nm) on erythrocytes infected by 10 genotypically distinct isolates of P. falciparum (isolate name in brackets), maintained in long-time in vitro culture and selected for expression of the PfEMP1 protein VAR2CSA by regular panning for IE adhesion to CSA (A–J). Individual data points, as well as the linear regression line (with 95% confidence limits) for data points <36 h are shown for each isolate. (TIF) [file pone.0045658.s004.tif]
