# Supplementary material for: The Density of Knobs on Plasmodium falciparum-Infected Erythrocytes Depends on Developmental Age and Varies among Isolates
Source: PLoS One. 2012 Sep 20;7(9):e45658. doi: 10.1371/journal.pone.0045658 (PMC3447797; doi:10.1371/journal.pone.0045658)
Supplement: Table S1 — Analysis of variance with test of linearity – Knob density and time since invasion among Ghanaian ex vivo parasite isolates (all time points). (DOCX) [file pone.0045658.s005.docx]

| **Isolate** |  | **SSq** | **DF** | **MSq** | **VR (F)** | **P(F)** |
| --- | --- | --- | --- | --- | --- | --- |
| GH1 | Regression  Dev. interval means  Within-interval residual | 12.20  125.35  191.61 | 1  1  17 | 12.20  125.35  11.27 | 1.08  11.12 | ≥0.05  **<0.005** |
| GH3 | Regression  Dev. interval means  Within-interval residual | 183.35  971.89  718.45 | 1  1  17 | 183.35  971.89  42.26 | 4.34  23.00 | ≥0.05  **<0.005** |
| GH4 | Regression  Dev. interval means  Within-interval residual | 95.61  225.57  1,926.81 | 1  1  17 | 95.61  225.57  113.34 | 0.84  1.99 | ≥0.05  ≥0.05 |
| GH5 * | Regression  Dev. interval means  Within-interval residual | 1,102.04  101.82  796.28 | 1  1  16 | 1,102.04  101.82  49.77 | 22.14  2.05 | **<0.005**  ≥0.05 |
| GH6 | Regression  Dev. interval means  Within-interval residual | 28.76  652.39  1,014.95 | 1  1  17 | 28.76  652.39  59.70 | 0.48  10.93 | ≥0.05  **<0.005** |
| GH7 * | Regression  Dev. interval means  Within-interval residual | 1,171.68  31.46  447.17 | 1  1  16 | 1,171.68  31.46  27.95 | 41.92  1.13 | **<0.005**  ≥0.05 |
| GH8 | Regression  Dev. interval means  Within-interval residual | 565.19  262.39  450.87 | 1  1  16 | 565.19  262.39  28.18 | 20.06  9.31 | **<0.005**  **<0.01** |
| GH9 | Regression  Dev. interval means  Within-interval residual | 52.81  1,399.65  974.37 | 1  1  18 | 52.81  1,399.65  54.13 | 0.98  25.86 | ≥0.05  **<0.005** |
| GH10 | Regression  Dev. interval means  Within-interval residual | 186.64  451.51  920.66 | 1  1  17 | 186.64  451.51  54.16 | 3.45  8.34 | ≥0.05  **<0.025** |
| GH11 | Regression  Dev. interval means  Within-interval residual | 1.10  275.83  1,403.92 | 1  1  16 | 1.10  275.83  87.74 | 0.01  3.14 | ≥0.05  ≥0.05 |
| GH14 | Regression  Dev. interval means  Within-interval residual | 10.01  475.19  607.02 | 1  1  17 | 10.01  475.19  35.70 | 0.28  13.31 | ≥0.05  **<0.01** |
| GH16 | Regression  Dev. interval means  Within-interval residual | 0.74  256.65  1,177.53 | 1  1  18 | 0.74  256.65  65.42 | 0.01  3.92 | ≥0.05  ≥0.05 |
| GH18 | Regression  Dev. interval means  Within-interval residual | 129.08  191.73  1,019.51 | 1  1  17 | 129.08  191.73  60.0 | 2.15  3.20 | ≥0.05  ≥0.05 |
| GH20 | Regression  Dev. interval means  Within-interval residual | 157.51  29.04  829.35 | 1  1  17 | 157.51  29.04  48.79 | 3.23  0.60 | ≥0.05  ≥0.05 |

* Isolates where the slope of the regression line was significant without evidence of departure from linearity are shaded gray.

## Supplementary Table 2. Analysis of variance with test of linearity – Knob density and time since invasion among Ghanaian ex vivo isolates (only time points < 36 h).

| **Isolate** |  | **SSq** | **DF** | **MSq** | **VR (F)** | **P(F)** |
| --- | --- | --- | --- | --- | --- | --- |
| GH1 * | Regression  Dev. interval means  Within-interval residual | 100.96  21.48  196.19 | 1  1  14 | 100.96  21.48  14.01 | 7.20  1.53 | **<0.025**  ≥0.05 |
| GH3 * | Regression  Dev. interval means  Within-interval residual | 764.58  32.64  901.43 | 1  1  12 | 764.58  32.64  75.12 | 10.18  0.43 | **<0.01**  ≥0.05 |
| GH4 * | Regression  Dev. interval means  Within-interval residual | 509.78  2.35  470.24 | 1  1  7 | 509.78  2.35  67.18 | 7.59  0.03 | **<0.05**  ≥0.05 |
| GH5 * | Regression  Dev. interval means  Within-interval residual | 629.90  41.00  1,012.43 | 1  1  15 | 629.90  41.00  67.50 | 9.33  0.61 | **<0.01**  ≥0.05 |
| GH6 * | Regression  Dev. interval means  Within-interval residual | 833.46  22.80  474.27 | 1  1  11 | 833.46  22.80  43.12 | 19.33  0.53 | **<0.005**  ≥0.05 |
| GH7 * | Regression  Dev. interval means  Within-interval residual | 1,029.44  5.74  581.78 | 1  1  15 | 1,029.44  5.74  38.79 | 26.54  0.15 | **<0.005**  ≥0.05 |
| GH8 * | Regression  Dev. interval means  Within-interval residual | 710.27  83.12  319.79 | 1  1  13 | 710.27  83.12  24.60 | 28.87  3.38 | **<0.005**  ≥0.05 |
| GH9 * | Regression  Dev. interval means  Within-interval residual | 1,347.08  3.89  886.36 | 1  1  12 | 1,347.08  3.89  73.86 | 18.24  0.05 | **<0.005**  ≥0.05 |
| GH10 | Regression  Dev. interval means  Within-interval residual | 77.41  1.48  754.33 | 1  1  9 | 77.41  1.48  83.81 | 0.92  0.01 | ≥0.05  ≥0.05 |
| GH11 * | Regression  Dev. interval means  Within-interval residual | 471.07  1.75  920.22 | 1  1  11 | 471.07  1.75  83.66 | 5.63  0.02 | **<0.05**  ≥0.05 |
| GH14 * | Regression  Dev. interval means  Within-interval residual | 645.96  6.71  67.10 | 1  1  9 | 645.96  6.71  7.45 | 86.69  0.90 | ***<0.005***  *≥0.05* |
| GH16 * | Regression  Dev. interval means  Within-interval residual | 332.66  22.47  418.10 | 1  1  13 | 322.66  22.47  32.16 | 10.03  0.70 | **<0.01**  ≥0.05 |
| GH18 | Regression  Dev. interval means  Within-interval residual | 3.33  46.82  717.72 | 1  1  15 | 3.33  46.82  47.85 | 0.07  0.98 | ≥0.05  ≥0.05 |
| GH20 | Regression  Dev. interval means  Within-interval residual | 0.18  99.48  533.85 | 1  1  14 | 0.18  99.48  38.13 | 0.00  2.61 | ≥0.05  ≥0.05 |

* Isolates where the slope of the regression line was significant without evidence of departure from linearity are shaded gray.

## Supplementary Table 3. Analysis of variance with test of linearity – Knob density and time since invasion among VAR2CSA-expressing long-term parasite isolates.

| **Isolate** |  | **SSq** | **DF** | **MSq** | **VR (F)** | **P(F)** |
| --- | --- | --- | --- | --- | --- | --- |
| FCR3 * | Regression  Dev. interval means  Within-interval residual | 8.72  0.25  25.5 | 1  1  16 | 8.72  0.25  1.59 | 5.47  0.16 | ***<0.05***  ≥0.05 |
| HB3 * | Regression  Dev. interval means  Within-interval residual | 30.53  8.81  36.35 | 1  1  17 | 30.53  8.81  2.14 | 14.27  4.12 | ***<0.005***  ≥0.05 |
| NF54 * | Regression  Dev. interval means  Within-interval residual | 32.94  0.62  42.79 | 1  1  17 | 32.94  0.62  2.52 | 13.08  0.24 | ***<0.005***  ≥0.05 |
| DP137 * | Regression  Dev. interval means  Within-interval residual | 16.34  0.32  47.56 | 1  1  15 | 16.34  0.32  3.17 | 5.15  0.10 | ***<0.05***  ≥0.05 |
| N4708 * | Regression  Dev. interval means  Within-interval residual | 8.29  0.01  11.17 | 1  1  17 | 8.29  0.01  0.66 | 12.63  0.01 | ***<0.005***  ≥0.05 |
| 7G8 | Regression  Dev. interval means  Within-interval residual | 1.67  1.74  15.09 | 1  1  19 | 1.67  1.74  0.79 | 2.11  2.19 | ≥0.05  ≥0.05 |
| 745 * | Regression  Dev. interval means  Within-interval residual | 21.64  2.92  40.86 | 1  1  17 | 21.64  2.92  2.40 | 9.01  0.95 | ***<0.01***  ≥0.05 |
| 748 * | Regression  Dev. interval means  Within-interval residual | 52.01  17.50  86.60 | 1  1  17 | 52.01  17.50  5.09 | 10.21  3.43 | ***<0.01***  ≥0.05 |
| 796 * | Regression  Dev. interval means  Within-interval residual | 46.78  1.55  89.08 | 1  1  15 | 46.78  1.55  5.94 | 7.88  0.26 | ***<0.025***  ≥0.05 |
| 7201 * | Regression  Dev. interval means  Within-interval residual | 27.82  6.18  69.03 | 1  1  15 | 27.82  6.18  4.60 | 6.05  1.34 | ***<0.025***  ≥0.05 |

* Isolates where the slope of the regression line was significant without evidence of departure from linearity are shaded gray.

## Supplementary Table 4. Analysis of variance with test of linearity – Knob density and time since invasion in isolate GH18 cultured in vitro for various length of time (only time points < 36 h).

| **Isolate** |  | **SSq** | **DF** | **MSq** | **VR (F)** | **P(F)** |
| --- | --- | --- | --- | --- | --- | --- |
| GH18,  1 day | Regression  Dev. interval means  Within-interval residual | 3.33  46.82  717.72 | 1  1  15 | 3.33  46.82  47.85 | 0.07  0.98 | ≥0.05  ≥0.05 |
| GH18,  8 wks | Regression  Dev. interval means  Within-interval residual | 162.60  156.76  471.53 | 1  1  12 | 162.60  156.76  39.29 | 4.14  3.99 | ≥0.05  ≥0.05 |
| GH18, 12 wks* | Regression  Dev. interval means  Within-interval residual | 333.69  0.578  328.17 | 1  1  11 | 333.69  0.578  29.83 | 11.19  0.02 | **<0.005**  ≥0.05 |

* Significant slope of the regression line without evidence of departure from linearity.

## Supplementary Table 5. Analysis of variance with test of linearity – Knob diameter and time since invasion among Ghanaian ex vivo parasite isolates (all time points).

| **Isolate** |  | **SSq** | **DF** | **MSq** | **VR (F)** | **P(F)** |
| --- | --- | --- | --- | --- | --- | --- |
| GH1 | Regression  Dev. interval means  Within-interval residual | 484.12  65.00  3,211.72 | 1  1  22 | 484.12  65.00  146.00 | 3.32  0.45 | ≥0.05  ≥0.05 |
| GH3 | Regression  Dev. interval means  Within-interval residual | 25.08  627.70  1,665.36 | 1  1  22 | 25.08  627.70  75.70 | 0.33  8.29 | ≥0.05  **<0.01** |
| GH4 | Regression  Dev. interval means  Within-interval residual | 253.11  424.46  1,682.47 | 1  1  22 | 253.11  424.46  76.48 | 3.31  5.55 | ≥0.05  **<0.05** |
| GH5 | Regression  Dev. interval means  Within-interval residual | 17.38  21.62  1,249.94 | 1  1  22 | 17.38  21.62  56.82 | 0.31  0.38 | ≥0.05  ≥0.05 |
| GH6 * | Regression  Dev. interval means  Within-interval residual | 3,319.36  85.29  2,144.34 | 1  1  22 | 3,319.36  85.29  97.47 | 34.06  0.88 | **<0.005**  ≥0.05 |
| GH7 | Regression  Dev. interval means  Within-interval residual | -  -  - | -  -  - | -  -  - | -  -  - | -  -  - |
| GH8 | Regression  Dev. interval means  Within-interval residual | 225.36  429.11  1,168.48 | 1  1  22 | 225.36  429.11  53.11 | 4.24  8.08 | ≥0.05  **<0.025** |
| GH9 | Regression  Dev. interval means  Within-interval residual | 93.43  618.02  1,258.89 | 1  1  22 | 93.43  618.02  57.22 | 1.63  10.80 | ≥0.05  **<0.005** |
| GH10 | Regression  Dev. interval means  Within-interval residual | 131.42  58.02  1,711.19 | 1  1  21 | 131.42  58.02  81.49 | 1.61  0.71 | ≥0.05  ≥0.05 |
| GH11 | Regression  Dev. interval means  Within-interval residual | 0.30  1,250.37  2,140.69 | 1  1  22 | 0.30  1,250.37  97.30 | 0.00  12.85 | ≥0.05  **<0.005** |
| GH14 | Regression  Dev. interval means  Within-interval residual | 25.72  54.93  2,204.09 | 1  1  20 | 25.72  54.93  104.96 | 0.25  0.52 | ≥0.05  ≥0.05 |
| GH16 | Regression  Dev. interval means  Within-interval residual | 152.77  358.96  2,016.22 | 1  1  22 | 152.77  358.96  91.65 | 1.67  3.92 | ≥0.05  ≥0.05 |
| GH18 | Regression  Dev. interval means  Within-interval residual | 1.21  25.87  996.66 | 1  1  21 | 1.21  25.87  47.46 | 0.03  0.55 | ≥0.05  ≥0.05 |
| GH20 * | Regression  Dev. interval means  Within-interval residual | 1,895.67  157.28  1,669.59 | 1  1  22 | 1,895.67  157.28  75.89 | 24.98  2.07 | **<0.005**  ≥0.05 |

* Isolates where the slope of the regression line was significant without evidence of departure from linearity are shaded gray.

## Supplementary Table 6. Analysis of variance with test of linearity – Knob height and time since invasion among Ghanaian ex vivo parasite isolates (all time points).

| **Isolate** |  | **SSq** | **DF** | **MSq** | **VR (F)** | **P(F)** |
| --- | --- | --- | --- | --- | --- | --- |
| GH1 | Regression  Dev. interval means  Within-interval residual | 0.01  1.51  21.13 | 1  1  22 | 0.01  1.51  0.96 | 0.01  1.57 | >0.05  >0.05 |
| GH3 | Regression  Dev. interval means  Within-interval residual | 1.27  2.83  7.10 | 1  1  21 | 1.27  2.83  0.34 | 3.74  8.37 | >0.05  **<0.01** |
| GH4 | Regression  Dev. interval means  Within-interval residual | 2.19  17.19  19.86 | 1  1  22 | 2.19  17.19  0.90 | 2.19  19.05 | >0.05  **<0.005** |
| GH5 | Regression  Dev. interval means  Within-interval residual | 0.11  10.17  16.10 | 1  1  21 | 0.11  10.17  0.77 | 0.14  13.27 | >0.05  **<0.005** |
| GH6 | Regression  Dev. interval means  Within-interval residual | 44.38  3.19  63.41 | 1  1  21 | 44.38  3.19  3.02 | 14.70  1.06 | **<0.005**  >0.05 |
| GH7 | Regression  Dev. interval means  Within-interval residual | -  -  - | -  -  - | -  -  - | -  -  - | -  -  - |
| GH8 | Regression  Dev. interval means  Within-interval residual | 4.72  1.39  8.82 | 1  1  22 | 4.72  1.39  0.40 | 11.77  3.46 | **<0.05**  >0.05 |
| GH9 | Regression  Dev. interval means  Within-interval residual | 0.34  6.41  21.07 | 1  1  22 | 0.34  6.41  0.96 | 0.36  6.69 | >0.05  **<0.025** |
| GH10 | Regression  Dev. interval means  Within-interval residual | 4.48  45.73  25.89 | 1  1  19 | 4.48  45.73  1.36 | 3.29  33.56 | >0.05  **<0.005** |
| GH11 | Regression  Dev. interval means  Within-interval residual | 1.83  9.69  99.49 | 1  1  21 | 1.83  9.69  4.74 | 0.39  2.05 | >0.05  >0.05 |
| GH14 | Regression  Dev. interval means  Within-interval residual | 6.47  0.31  58.80 | 1  1  20 | 6.47  0.31  2.94 | 2.20  0.11 | >0.05  >0.05 |
| GH16 | Regression  Dev. interval means  Within-interval residual | 2.04  0.00  25.77 | 1  1  21 | 2.04  0.00  1.23 | 1.67  0.00 | >0.05  >0.05 |
| GH18 * | Regression  Dev. interval means  Within-interval residual | 0.02  1.21  7.18 | 1  1  16 | 0.02  1.21  0.45 | 0.04  2.70 | >0.05  >0.05 |
| GH20 * | Regression  Dev. interval means  Within-interval residual | 2.49  7.27  13.05 | 1  1  22 | 2.49  7.27  0.59 | 4.19  12.25 | >0.05  **<0.005** |

* Isolates where the slope of the regression line was significant without evidence of departure from linearity are shaded gray.

## Supplementary Table 7. Analysis of variance with test of linearity - Knob diameter and time since invasion among VAR2CSA-expressing long-term parasite isolates.

| **Isolate** |  | **SSq** | **DF** | **MSq** | **VR (F)** | **P(F)** |
| --- | --- | --- | --- | --- | --- | --- |
| FCR3 | Regression  Dev. interval means  Within-interval residual | 2,010.81  1,608.88  9,163.89 | 1  1  35 | 2,010.81  1,608.88  261.825 | 7.68  6.14 | **<0.01**  **<0.025** |
| HB3 | Regression  Dev. interval means  Within-interval residual | 1.98  3,170.9  5,986.6 | 1  1  33 | 1.982  3,170.92  193.12 | 0.01  16.42 | ≥0.05  **<0.005** |
| NF54 | Regression  Dev. interval means  Within-interval residual | 781.95  1,662.48  16,081.1 | 1  1  31 | 781.95  1,662.48  518.75 | 1.51  3.20 | ≥0.05  ≥0.05 |
| DP137 | Regression  Dev. interval means  Within-interval residual | 6,095.47  5,200.23  7,134.28 | 1  1  29 | 6,095.47  5,200.23  246.01 | 24.78  21.14 | **<0.005**  **<0.005** |
| N4708 * | Regression  Dev. interval means  Within-interval residual | 29,745.3  2.31  20,093.3 | 1  1  35 | 29,745.3  2.31  574.09 | 51.81  0.00 | **<0.005**  ≥0.05 |
| 7G8 | Regression  Dev. interval means  Within-interval residual | 612.48  1,195.31  5,135.69 | 1  1  35 | 612.480  1,195.31  146.73 | 4.17  8.15 | ≥0.05  **<0.025** |
| 745 | Regression  Dev. interval means  Within-interval residual | 504.37  1,178.64  16,769.3 | 1  1  31 | 504.37  1,178.64  540.94 | 0.932  2.178 | ≥0.05  ≥0.05 |
| 748 | Regression  Dev. interval means  Within-interval residual | 1,789.91  12,225.4  13,310.1 | 1  1  35 | 1,789.91  12,225.35  380.29 | 4.707  32.146 | **<0.05**  **<0.005** |
| 796 | Regression  Dev. interval means  Within-interval residual | 135.25  529.54  7,096.93 | 1  1  35 | 135.25  529.54  202.77 | 0.67  2.61 | ≥0.05  ≥0.05 |
| 7201 | Regression  Dev. interval means  Within-interval residual | 5,999.31  2,105.32  4,578.24 | 1  1  35 | 5,999.31  2,105.32  130.81 | 45.864  16.095 | **<0.005**  **<0.005** |

* Significant slope of the regression line without evidence of departure from linearity.

## Supplementary Table 8. Analysis of variance with test of linearity - Knob height and time since invasion among VAR2CSA-expressing long-term parasite isolates.

| **Isolate** |  | **SSq** | **DF** | **MSq** | **VR (F)** | **P(F)** |
| --- | --- | --- | --- | --- | --- | --- |
| FCR3 * | Regression  Dev. interval means  Within-interval residual | 42.38  1.73  42.16 | 1  1  21 | 42.38  1.73  2.01 | 21.11  0.86 | **<0.005**  >0.05 |
| HB3 | Regression  Dev. interval means  Within-interval residual | 7.65  12.41  13.75 | 1  1  21 | 7.65  12.41  0.65 | 11.68  18.97 | **<0.001**  **<0.001** |
| NF54 | Regression  Dev. interval means  Within-interval residual | 8.09  10.88  26.60 | 1  1  17 | 8.09  10.88  1.56 | 5.17  6.96 | **<0.05**  **<0.025** |
| DP137 | Regression  Dev. interval means  Within-interval residual | 6.87  15.24  28.88 | 1  1  16 | 6.87  15.24  1.80 | 3.81  8.45 | >0.05  **<0.01** |
| N4708 * | Regression  Dev. interval means  Within-interval residual | 32.50  1.95  73.75 | 1  1  21 | 32.50  1.95  3.51 | 9.25  0.56 | **<0.01**  >0.05 |
| 7G8 | Regression  Dev. interval means  Within-interval residual | 0.10  0.17  16.56 | 1  1  21 | 0.10  0.17  0.79 | 0.21  0.22 | >0.05  >0.05 |
| 745 | Regression  Dev. interval means  Within-interval residual | 0.67  1.61  75.41 | 1  1  19 | 0.67  1.61  3.97 | 0.17  0.40 | >0.05  >0.05 |
| 748 | Regression  Dev. interval means  Within-interval residual | 48.06  13.67  148.39 | 1  1  22 | 48.06  13.67  6.75 | 7.12  2.03 | **<0.025**  >0.05 |
| 796 | Regression  Dev. interval means  Within-interval residual | 3.99  3.94  70.05 | 1  1  19 | 3.99  3.94  3.69 | 1.08  1.07 | >0.05  >0.05 |
| 7201 * | Regression  Dev. interval means  Within-interval residual | 72.14  104.48  90.68 | 1  1  22 | 72.14  104.48  4.21 | 17.50  25.35 | **<0.005**  **<0.005** |

* Isolates where the slope of the regression line was significant without evidence of departure from linearity are shaded gray.
