# Supplementary material for: The Density of Knobs on Plasmodium falciparum-Infected Erythrocytes Depends on Developmental Age and Varies among Isolates
Source: PLoS One. 2012 Sep 20;7(9):e45658. doi: 10.1371/journal.pone.0045658 (PMC3447797; doi:10.1371/journal.pone.0045658)
Supplement: Table S2 — Analysis of variance with test of linearity – Knob density and time since invasion among Ghanaian ex vivo isolates (only time points <36 h). (DOCX) [file pone.0045658.s006.docx]

| **Isolate** |  | **SSq** | **DF** | **MSq** | **VR (F)** | **P(F)** |
| --- | --- | --- | --- | --- | --- | --- |
| GH1 * | Regression  Dev. interval means  Within-interval residual | 100.96  21.48  196.19 | 1  1  14 | 100.96  21.48  14.01 | 7.20  1.53 | **<0.025**  ≥0.05 |
| GH3 * | Regression  Dev. interval means  Within-interval residual | 764.58  32.64  901.43 | 1  1  12 | 764.58  32.64  75.12 | 10.18  0.43 | **<0.01**  ≥0.05 |
| GH4 * | Regression  Dev. interval means  Within-interval residual | 509.78  2.35  470.24 | 1  1  7 | 509.78  2.35  67.18 | 7.59  0.03 | **<0.05**  ≥0.05 |
| GH5 * | Regression  Dev. interval means  Within-interval residual | 629.90  41.00  1,012.43 | 1  1  15 | 629.90  41.00  67.50 | 9.33  0.61 | **<0.01**  ≥0.05 |
| GH6 * | Regression  Dev. interval means  Within-interval residual | 833.46  22.80  474.27 | 1  1  11 | 833.46  22.80  43.12 | 19.33  0.53 | **<0.005**  ≥0.05 |
| GH7 * | Regression  Dev. interval means  Within-interval residual | 1,029.44  5.74  581.78 | 1  1  15 | 1,029.44  5.74  38.79 | 26.54  0.15 | **<0.005**  ≥0.05 |
| GH8 * | Regression  Dev. interval means  Within-interval residual | 710.27  83.12  319.79 | 1  1  13 | 710.27  83.12  24.60 | 28.87  3.38 | **<0.005**  ≥0.05 |
| GH9 * | Regression  Dev. interval means  Within-interval residual | 1,347.08  3.89  886.36 | 1  1  12 | 1,347.08  3.89  73.86 | 18.24  0.05 | **<0.005**  ≥0.05 |
| GH10 | Regression  Dev. interval means  Within-interval residual | 77.41  1.48  754.33 | 1  1  9 | 77.41  1.48  83.81 | 0.92  0.01 | ≥0.05  ≥0.05 |
| GH11 * | Regression  Dev. interval means  Within-interval residual | 471.07  1.75  920.22 | 1  1  11 | 471.07  1.75  83.66 | 5.63  0.02 | **<0.05**  ≥0.05 |
| GH14 * | Regression  Dev. interval means  Within-interval residual | 645.96  6.71  67.10 | 1  1  9 | 645.96  6.71  7.45 | 86.69  0.90 | ***<0.005***  *≥0.05* |
| GH16 * | Regression  Dev. interval means  Within-interval residual | 332.66  22.47  418.10 | 1  1  13 | 322.66  22.47  32.16 | 10.03  0.70 | **<0.01**  ≥0.05 |
| GH18 | Regression  Dev. interval means  Within-interval residual | 3.33  46.82  717.72 | 1  1  15 | 3.33  46.82  47.85 | 0.07  0.98 | ≥0.05  ≥0.05 |
| GH20 | Regression  Dev. interval means  Within-interval residual | 0.18  99.48  533.85 | 1  1  14 | 0.18  99.48  38.13 | 0.00  2.61 | ≥0.05  ≥0.05 |

* Isolates where the slope of the regression line was significant without evidence of departure from linearity are shaded gray.
