# Supplementary material for: The Density of Knobs on Plasmodium falciparum-Infected Erythrocytes Depends on Developmental Age and Varies among Isolates
Source: PLoS One. 2012 Sep 20;7(9):e45658. doi: 10.1371/journal.pone.0045658 (PMC3447797; doi:10.1371/journal.pone.0045658)
Supplement: Table S3 — Analysis of variance with test of linearity – Knob density and time since invasion among VAR2CSA-expressing long-term parasite isolates. (DOCX) [file pone.0045658.s007.docx]

| **Isolate** |  | **SSq** | **DF** | **MSq** | **VR (F)** | **P(F)** |
| --- | --- | --- | --- | --- | --- | --- |
| FCR3 * | Regression  Dev. interval means  Within-interval residual | 8.72  0.25  25.5 | 1  1  16 | 8.72  0.25  1.59 | 5.47  0.16 | ***<0.05***  ≥0.05 |
| HB3 * | Regression  Dev. interval means  Within-interval residual | 30.53  8.81  36.35 | 1  1  17 | 30.53  8.81  2.14 | 14.27  4.12 | ***<0.005***  ≥0.05 |
| NF54 * | Regression  Dev. interval means  Within-interval residual | 32.94  0.62  42.79 | 1  1  17 | 32.94  0.62  2.52 | 13.08  0.24 | ***<0.005***  ≥0.05 |
| DP137 * | Regression  Dev. interval means  Within-interval residual | 16.34  0.32  47.56 | 1  1  15 | 16.34  0.32  3.17 | 5.15  0.10 | ***<0.05***  ≥0.05 |
| N4708 * | Regression  Dev. interval means  Within-interval residual | 8.29  0.01  11.17 | 1  1  17 | 8.29  0.01  0.66 | 12.63  0.01 | ***<0.005***  ≥0.05 |
| 7G8 | Regression  Dev. interval means  Within-interval residual | 1.67  1.74  15.09 | 1  1  19 | 1.67  1.74  0.79 | 2.11  2.19 | ≥0.05  ≥0.05 |
| 745 * | Regression  Dev. interval means  Within-interval residual | 21.64  2.92  40.86 | 1  1  17 | 21.64  2.92  2.40 | 9.01  0.95 | ***<0.01***  ≥0.05 |
| 748 * | Regression  Dev. interval means  Within-interval residual | 52.01  17.50  86.60 | 1  1  17 | 52.01  17.50  5.09 | 10.21  3.43 | ***<0.01***  ≥0.05 |
| 796 * | Regression  Dev. interval means  Within-interval residual | 46.78  1.55  89.08 | 1  1  15 | 46.78  1.55  5.94 | 7.88  0.26 | ***<0.025***  ≥0.05 |
| 7201 * | Regression  Dev. interval means  Within-interval residual | 27.82  6.18  69.03 | 1  1  15 | 27.82  6.18  4.60 | 6.05  1.34 | ***<0.025***  ≥0.05 |

* Isolates where the slope of the regression line was significant without evidence of departure from linearity are shaded gray.
