# Supplementary material for: The Density of Knobs on Plasmodium falciparum-Infected Erythrocytes Depends on Developmental Age and Varies among Isolates
Source: PLoS One. 2012 Sep 20;7(9):e45658. doi: 10.1371/journal.pone.0045658 (PMC3447797; doi:10.1371/journal.pone.0045658)
Supplement: Table S4 — Analysis of variance with test of linearity – Knob density and time since invasion in isolate GH18 cultured in vitro for various length of time (only time points <36 h). (DOCX) [file pone.0045658.s008.docx]

| **Isolate** |  | **SSq** | **DF** | **MSq** | **VR (F)** | **P(F)** |
| --- | --- | --- | --- | --- | --- | --- |
| GH18,  1 day | Regression  Dev. interval means  Within-interval residual | 3.33  46.82  717.72 | 1  1  15 | 3.33  46.82  47.85 | 0.07  0.98 | ≥0.05  ≥0.05 |
| GH18,  8 wks | Regression  Dev. interval means  Within-interval residual | 162.60  156.76  471.53 | 1  1  12 | 162.60  156.76  39.29 | 4.14  3.99 | ≥0.05  ≥0.05 |
| GH18, 12 wks* | Regression  Dev. interval means  Within-interval residual | 333.69  0.578  328.17 | 1  1  11 | 333.69  0.578  29.83 | 11.19  0.02 | **<0.005**  ≥0.05 |

* Significant slope of the regression line without evidence of departure from linearity.
