# Supplementary material for: The Density of Knobs on Plasmodium falciparum-Infected Erythrocytes Depends on Developmental Age and Varies among Isolates
Source: PLoS One. 2012 Sep 20;7(9):e45658. doi: 10.1371/journal.pone.0045658 (PMC3447797; doi:10.1371/journal.pone.0045658)
Supplement: Table S5 — Analysis of variance with test of linearity – Knob diameter and time since invasion among Ghanaian ex vivo parasite isolates (all time points). (DOCX) [file pone.0045658.s009.docx]

| **Isolate** |  | **SSq** | **DF** | **MSq** | **VR (F)** | **P(F)** |
| --- | --- | --- | --- | --- | --- | --- |
| GH1 | Regression  Dev. interval means  Within-interval residual | 484.12  65.00  3,211.72 | 1  1  22 | 484.12  65.00  146.00 | 3.32  0.45 | ≥0.05  ≥0.05 |
| GH3 | Regression  Dev. interval means  Within-interval residual | 25.08  627.70  1,665.36 | 1  1  22 | 25.08  627.70  75.70 | 0.33  8.29 | ≥0.05  **<0.01** |
| GH4 | Regression  Dev. interval means  Within-interval residual | 253.11  424.46  1,682.47 | 1  1  22 | 253.11  424.46  76.48 | 3.31  5.55 | ≥0.05  **<0.05** |
| GH5 | Regression  Dev. interval means  Within-interval residual | 17.38  21.62  1,249.94 | 1  1  22 | 17.38  21.62  56.82 | 0.31  0.38 | ≥0.05  ≥0.05 |
| GH6 * | Regression  Dev. interval means  Within-interval residual | 3,319.36  85.29  2,144.34 | 1  1  22 | 3,319.36  85.29  97.47 | 34.06  0.88 | **<0.005**  ≥0.05 |
| GH7 | Regression  Dev. interval means  Within-interval residual | -  -  - | -  -  - | -  -  - | -  -  - | -  -  - |
| GH8 | Regression  Dev. interval means  Within-interval residual | 225.36  429.11  1,168.48 | 1  1  22 | 225.36  429.11  53.11 | 4.24  8.08 | ≥0.05  **<0.025** |
| GH9 | Regression  Dev. interval means  Within-interval residual | 93.43  618.02  1,258.89 | 1  1  22 | 93.43  618.02  57.22 | 1.63  10.80 | ≥0.05  **<0.005** |
| GH10 | Regression  Dev. interval means  Within-interval residual | 131.42  58.02  1,711.19 | 1  1  21 | 131.42  58.02  81.49 | 1.61  0.71 | ≥0.05  ≥0.05 |
| GH11 | Regression  Dev. interval means  Within-interval residual | 0.30  1,250.37  2,140.69 | 1  1  22 | 0.30  1,250.37  97.30 | 0.00  12.85 | ≥0.05  **<0.005** |
| GH14 | Regression  Dev. interval means  Within-interval residual | 25.72  54.93  2,204.09 | 1  1  20 | 25.72  54.93  104.96 | 0.25  0.52 | ≥0.05  ≥0.05 |
| GH16 | Regression  Dev. interval means  Within-interval residual | 152.77  358.96  2,016.22 | 1  1  22 | 152.77  358.96  91.65 | 1.67  3.92 | ≥0.05  ≥0.05 |
| GH18 | Regression  Dev. interval means  Within-interval residual | 1.21  25.87  996.66 | 1  1  21 | 1.21  25.87  47.46 | 0.03  0.55 | ≥0.05  ≥0.05 |
| GH20 * | Regression  Dev. interval means  Within-interval residual | 1,895.67  157.28  1,669.59 | 1  1  22 | 1,895.67  157.28  75.89 | 24.98  2.07 | **<0.005**  ≥0.05 |

* Isolates where the slope of the regression line was significant without evidence of departure from linearity are shaded gray.
