# Supplementary material for: The Density of Knobs on Plasmodium falciparum-Infected Erythrocytes Depends on Developmental Age and Varies among Isolates
Source: PLoS One. 2012 Sep 20;7(9):e45658. doi: 10.1371/journal.pone.0045658 (PMC3447797; doi:10.1371/journal.pone.0045658)
Supplement: Table S6 — Analysis of variance with test of linearity – Knob height and time since invasion among Ghanaian ex vivo parasite isolates (all time points). (DOCX) [file pone.0045658.s010.docx]

| **Isolate** |  | **SSq** | **DF** | **MSq** | **VR (F)** | **P(F)** |
| --- | --- | --- | --- | --- | --- | --- |
| GH1 | Regression  Dev. interval means  Within-interval residual | 0.01  1.51  21.13 | 1  1  22 | 0.01  1.51  0.96 | 0.01  1.57 | >0.05  >0.05 |
| GH3 | Regression  Dev. interval means  Within-interval residual | 1.27  2.83  7.10 | 1  1  21 | 1.27  2.83  0.34 | 3.74  8.37 | >0.05  **<0.01** |
| GH4 | Regression  Dev. interval means  Within-interval residual | 2.19  17.19  19.86 | 1  1  22 | 2.19  17.19  0.90 | 2.19  19.05 | >0.05  **<0.005** |
| GH5 | Regression  Dev. interval means  Within-interval residual | 0.11  10.17  16.10 | 1  1  21 | 0.11  10.17  0.77 | 0.14  13.27 | >0.05  **<0.005** |
| GH6 | Regression  Dev. interval means  Within-interval residual | 44.38  3.19  63.41 | 1  1  21 | 44.38  3.19  3.02 | 14.70  1.06 | **<0.005**  >0.05 |
| GH7 | Regression  Dev. interval means  Within-interval residual | -  -  - | -  -  - | -  -  - | -  -  - | -  -  - |
| GH8 | Regression  Dev. interval means  Within-interval residual | 4.72  1.39  8.82 | 1  1  22 | 4.72  1.39  0.40 | 11.77  3.46 | **<0.05**  >0.05 |
| GH9 | Regression  Dev. interval means  Within-interval residual | 0.34  6.41  21.07 | 1  1  22 | 0.34  6.41  0.96 | 0.36  6.69 | >0.05  **<0.025** |
| GH10 | Regression  Dev. interval means  Within-interval residual | 4.48  45.73  25.89 | 1  1  19 | 4.48  45.73  1.36 | 3.29  33.56 | >0.05  **<0.005** |
| GH11 | Regression  Dev. interval means  Within-interval residual | 1.83  9.69  99.49 | 1  1  21 | 1.83  9.69  4.74 | 0.39  2.05 | >0.05  >0.05 |
| GH14 | Regression  Dev. interval means  Within-interval residual | 6.47  0.31  58.80 | 1  1  20 | 6.47  0.31  2.94 | 2.20  0.11 | >0.05  >0.05 |
| GH16 | Regression  Dev. interval means  Within-interval residual | 2.04  0.00  25.77 | 1  1  21 | 2.04  0.00  1.23 | 1.67  0.00 | >0.05  >0.05 |
| GH18 * | Regression  Dev. interval means  Within-interval residual | 0.02  1.21  7.18 | 1  1  16 | 0.02  1.21  0.45 | 0.04  2.70 | >0.05  >0.05 |
| GH20 * | Regression  Dev. interval means  Within-interval residual | 2.49  7.27  13.05 | 1  1  22 | 2.49  7.27  0.59 | 4.19  12.25 | >0.05  **<0.005** |

* Isolates where the slope of the regression line was significant without evidence of departure from linearity are shaded gray.
