# Supplementary material for: The Density of Knobs on Plasmodium falciparum-Infected Erythrocytes Depends on Developmental Age and Varies among Isolates
Source: PLoS One. 2012 Sep 20;7(9):e45658. doi: 10.1371/journal.pone.0045658 (PMC3447797; doi:10.1371/journal.pone.0045658)
Supplement: Table S7 — Analysis of variance with test of linearity - Knob diameter and time since invasion among VAR2CSA-expressing long-term parasite isolates. (DOCX) [file pone.0045658.s011.docx]

| **Isolate** |  | **SSq** | **DF** | **MSq** | **VR (F)** | **P(F)** |
| --- | --- | --- | --- | --- | --- | --- |
| FCR3 | Regression  Dev. interval means  Within-interval residual | 2,010.81  1,608.88  9,163.89 | 1  1  35 | 2,010.81  1,608.88  261.825 | 7.68  6.14 | **<0.01**  **<0.025** |
| HB3 | Regression  Dev. interval means  Within-interval residual | 1.98  3,170.9  5,986.6 | 1  1  33 | 1.982  3,170.92  193.12 | 0.01  16.42 | ≥0.05  **<0.005** |
| NF54 | Regression  Dev. interval means  Within-interval residual | 781.95  1,662.48  16,081.1 | 1  1  31 | 781.95  1,662.48  518.75 | 1.51  3.20 | ≥0.05  ≥0.05 |
| DP137 | Regression  Dev. interval means  Within-interval residual | 6,095.47  5,200.23  7,134.28 | 1  1  29 | 6,095.47  5,200.23  246.01 | 24.78  21.14 | **<0.005**  **<0.005** |
| N4708 * | Regression  Dev. interval means  Within-interval residual | 29,745.3  2.31  20,093.3 | 1  1  35 | 29,745.3  2.31  574.09 | 51.81  0.00 | **<0.005**  ≥0.05 |
| 7G8 | Regression  Dev. interval means  Within-interval residual | 612.48  1,195.31  5,135.69 | 1  1  35 | 612.480  1,195.31  146.73 | 4.17  8.15 | ≥0.05  **<0.025** |
| 745 | Regression  Dev. interval means  Within-interval residual | 504.37  1,178.64  16,769.3 | 1  1  31 | 504.37  1,178.64  540.94 | 0.932  2.178 | ≥0.05  ≥0.05 |
| 748 | Regression  Dev. interval means  Within-interval residual | 1,789.91  12,225.4  13,310.1 | 1  1  35 | 1,789.91  12,225.35  380.29 | 4.707  32.146 | **<0.05**  **<0.005** |
| 796 | Regression  Dev. interval means  Within-interval residual | 135.25  529.54  7,096.93 | 1  1  35 | 135.25  529.54  202.77 | 0.67  2.61 | ≥0.05  ≥0.05 |
| 7201 | Regression  Dev. interval means  Within-interval residual | 5,999.31  2,105.32  4,578.24 | 1  1  35 | 5,999.31  2,105.32  130.81 | 45.864  16.095 | **<0.005**  **<0.005** |

* Significant slope of the regression line without evidence of departure from linearity.
