# Supplementary material for: The Density of Knobs on Plasmodium falciparum-Infected Erythrocytes Depends on Developmental Age and Varies among Isolates
Source: PLoS One. 2012 Sep 20;7(9):e45658. doi: 10.1371/journal.pone.0045658 (PMC3447797; doi:10.1371/journal.pone.0045658)
Supplement: Table S8 — Analysis of variance with test of linearity - Knob height and time since invasion among VAR2CSA-expressing long-term parasite isolates. (DOCX) [file pone.0045658.s012.docx]

| **Isolate** |  | **SSq** | **DF** | **MSq** | **VR (F)** | **P(F)** |
| --- | --- | --- | --- | --- | --- | --- |
| FCR3 * | Regression  Dev. interval means  Within-interval residual | 42.38  1.73  42.16 | 1  1  21 | 42.38  1.73  2.01 | 21.11  0.86 | **<0.005**  >0.05 |
| HB3 | Regression  Dev. interval means  Within-interval residual | 7.65  12.41  13.75 | 1  1  21 | 7.65  12.41  0.65 | 11.68  18.97 | **<0.001**  **<0.001** |
| NF54 | Regression  Dev. interval means  Within-interval residual | 8.09  10.88  26.60 | 1  1  17 | 8.09  10.88  1.56 | 5.17  6.96 | **<0.05**  **<0.025** |
| DP137 | Regression  Dev. interval means  Within-interval residual | 6.87  15.24  28.88 | 1  1  16 | 6.87  15.24  1.80 | 3.81  8.45 | >0.05  **<0.01** |
| N4708 * | Regression  Dev. interval means  Within-interval residual | 32.50  1.95  73.75 | 1  1  21 | 32.50  1.95  3.51 | 9.25  0.56 | **<0.01**  >0.05 |
| 7G8 | Regression  Dev. interval means  Within-interval residual | 0.10  0.17  16.56 | 1  1  21 | 0.10  0.17  0.79 | 0.21  0.22 | >0.05  >0.05 |
| 745 | Regression  Dev. interval means  Within-interval residual | 0.67  1.61  75.41 | 1  1  19 | 0.67  1.61  3.97 | 0.17  0.40 | >0.05  >0.05 |
| 748 | Regression  Dev. interval means  Within-interval residual | 48.06  13.67  148.39 | 1  1  22 | 48.06  13.67  6.75 | 7.12  2.03 | **<0.025**  >0.05 |
| 796 | Regression  Dev. interval means  Within-interval residual | 3.99  3.94  70.05 | 1  1  19 | 3.99  3.94  3.69 | 1.08  1.07 | >0.05  >0.05 |
| 7201 * | Regression  Dev. interval means  Within-interval residual | 72.14  104.48  90.68 | 1  1  22 | 72.14  104.48  4.21 | 17.50  25.35 | **<0.005**  **<0.005** |

* Isolates where the slope of the regression line was significant without evidence of departure from linearity are shaded gray.
